# Amelioration of the reduced antinociceptive effect of morphine in the unpredictable chronic mild stress model mice by noradrenalin but not serotonin reuptake inhibitors

**DOI:** 10.1186/s12990-015-0051-0

**Published:** 2015-08-11

**Authors:** Soichiro Ide, Hiroshi Satoyoshi, Masabumi Minami, Masamichi Satoh

**Affiliations:** Department of Pharmacology, Graduate School of Pharmaceutical Sciences, Hokkaido University, Sapporo, Japan; Addictive Substance Project, Tokyo Metropolitan Institute of Medical Science, Tokyo, Japan; Graduate School of Pharmaceutical Sciences, Kyoto University, Kyoto, Japan

**Keywords:** Opioid, Unpredictable chronic mild stress, Pain, Analgesia, Morphine, Tramadol, Noradrenalin reuptake inhibitor, Serotonin reuptake inhibitor

## Abstract

**Background:**

Although alterations in not only the pain sensitivity but also the analgesic effects of opioids have been reported under conditions of stress, the influence of unpredictable chronic mild stress (UCMS) on the antinociceptive effects of opioid analgesics remains to be fully investigated. The present study examined the influence of UCMS on the thermal pain sensitivity and antinociceptive effects of two opioid analgesics, morphine (an agonist of opioid receptors) and tramadol (an agonist of μ-opioid receptor and an inhibitor of both noradrenaline and serotonin transporters). We also examined the effects of pretreatment with maprotiline (a noradrenaline reuptake inhibitor) and escitalopram (a serotonin reuptake inhibitor) on the antinociceptive action of morphine in mice under an UCMS condition.

**Results:**

Unpredictable chronic mild stress did not affect the basal thermal pain sensitivity in a mouse hot-plate test. Although morphine dose-dependently induced thermal antinociceptive effects under both the UCMS and non-stress conditions, the thermal antinociceptive effect of 3 mg/kg morphine under the UCMS condition was significantly lower than under the non-stressed condition. Unlike the case with morphine, we observed no significant difference in the thermal antinociceptive effect of tramadol between the UCMS and non-stress conditions. Furthermore, the reduced thermal antinociceptive effect of 3 mg/kg morphine under the UCMS condition was significantly ameliorated by pretreatment with 10 mg/kg maprotiline but not 3 mg/kg escitalopram. Pretreatment with neither maprotiline nor escitalopram alone was associated with an antinociceptive effect under either condition.

**Conclusions:**

We demonstrated that the antinociceptive effect of morphine but not tramadol was reduced in mice that had experienced UCMS. The reduced antinociceptive effect of morphine under the UCMS condition was ameliorated by pretreatment with maprotiline but not escitalopram. These results suggest that the reduced antinociceptive effects of morphine under conditions of chronic stress may be ameliorated by activation of the noradrenergic but not the serotonergic system.

## Background

Endogenous opioid systems play important roles in the modulation of pain sensitivity and stress responses. On the other hand, various types of stressors have been known to alter not only pain sensitivity but also the analgesic effects of opioids [1]. Several studies have demonstrated the potentiation of the antinociceptive effects of opioid analgesics by acute stress, such as restraint stress and cold-water swim stress [1–3], whereas other reports have shown the attenuation of the antinociceptive effect of morphine by chronic stress, such as chronic restraint stress and chronic cold-water swim stress [4, 5]. However, the mechanisms underlying the reduced antinociceptive effect of morphine under conditions of chronic stress remain to be elucidated.

Opioid analgesics, such as morphine and tramadol, are used widely for the treatment of moderate to severe pain. Morphine is the most widely used ancient opioid analgesic and is known to exert analgesic effects by acting on μ-opioid receptors [6–8]. Tramadol, a racemic mixture of two enantiomers, is one of the most widely used weak opioid analgesics. Its major metabolite, (+)-*O*-desmethyltramadol (M1), acts on opioid receptors as an agonist [9–11]. Furthermore, (+)- and (−)-tramadol enantiomers inhibit neuronal reuptakes of serotonin and noradrenaline, respectively [12–14]. We previously showed that the thermal antinociceptive effects of tramadol were mediated primarily by its actions on μ-opioid receptors and noradrenalin transporters but not on serotonin transporters [9]. Because little is known about the influences of monoaminetransporters on the analgesic effects of opioids under conditions of chronic stress, we compared the antinociceptive effects of morphine and tramadol in mice under conditions of unpredictable chronic mild stress (UCMS).

## Results

**Fig. 1:**
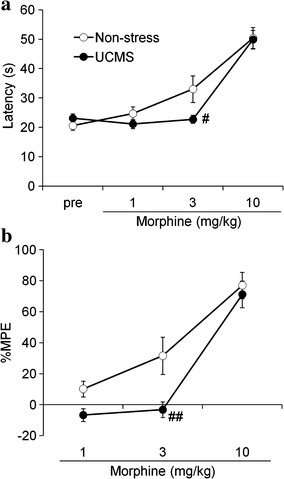
Thermal antinociceptive effects of morphine under the UCMS condition. The effects of morphine on thermal nociceptive latency (**a**) and %MPE (**b**) in a hot-plate test under the UCMS (*closed circles* *n* = 15) and non-stress (*open circles* *n* = 13) conditions. Intraperitoneal morphine was repetitively administered to the mice at doses of 1, 2, and 7 mg/kg, for cumulative doses of 1, 3, and 10 mg/kg, respectively. ^##^ *p* < 0.01, ^#^ *p* < 0.05, significantly differed from the non-stress condition. Data are expressed as mean ± SEM.

Dose–response relationships in the thermal antinociceptive effects of morphine were examined under the UCMS and non-stress conditions (Fig. 1). We found no significant differences in the hot-plate latencies under the UCMS (23.1 ± 1.5 s) and non-stress (20.6 ± 1.6 s) conditions before drug treatment (Fig. 1a), indicating that the UCMS procedure used in the present study did not affect the basal thermal pain sensitivity. Morphine dose-dependently produced thermal antinociceptive effects in a hot-plate test under both UCMS and non-stress conditions. A two-way repeated-measures analysis of variance (ANOVA) revealed that the thermal antinociceptive effects of morphine (%MPE) significantly differed under the UCMS and non-stress conditions (stress, *F*_1,26_ = 5.72, *p* = 0.024; dose, *F*_2,52_ = 78.22, *p* < 0.001; stress × dose interaction, *F*_2,52_ = 2.81, *p* = 0.070; Fig. 1b). The thermal antinociceptive effect of 3 mg/kg morphine was significantly lower under the UCMS than the non-stress condition (*p* < 0.01, Sidak’s multiple comparisons post hoc test). In contrast, we observed no significant differences between these two conditions in the antinociceptive effects of lower (1 mg/kg) and higher (10 mg/kg) doses of morphine.

**Fig. 2:**
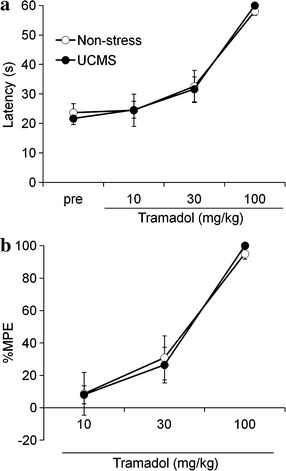
Thermal antinociceptive effects of tramadol under the UCMS condition. The effects of tramadol on thermal nociceptive latency (**a**) and %MPE (**b**) in a hot-plate test under the UCMS (*closed circles* *n* = 8) and non-stress (*open circles* *n* = 8) conditions. Intraperitoneal tramadol was repetitively administered to the mice at doses of 10, 20, and 70 mg/kg, for cumulative doses of 10, 30, and 100 mg/kg, respectively. Data are expressed as mean ± SEM.

Next, dose–response relationships in the thermal antinociceptive effects of tramadol were examined under the UCMS and non-stress conditions (Fig. 2). We found no significant difference in the hot-plate latency between the UCMS (21.7 ± 2.0 s) and non-stress (23.7 ± 3.1 s) conditions before drug-treatment (Fig. 2a). Tramadol dose-dependently produced thermal antinociceptive effects in a hot-plate test under both the UCMS and non-stress conditions. Unlike the case of morphine, no significant differences in the antinociceptive effects of tramadol were observed under the UCMS and non-stress conditions (Fig. 2b).

**Fig. 3:**
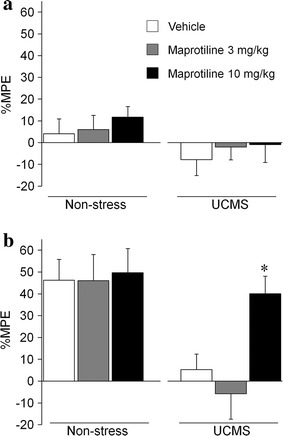
Effects of maprotiline on the antinociceptive effect of morphine under the UCMS condition. The effects of maprotiline on the thermal pain sensitivity (**a**) and the antinociceptive effect of morphine (3 mg/kg; **b**) in a hot-plate test under the UCMS (*white column* vehicle, *n* = 12; *gray column* maprotiline 3 mg/kg, *n* = 12; *black column* maprotiline 10 mg/kg, *n* = 14) and non-stress (*white column* vehicle, *n* = 14; *gray column* maprotiline 3 mg/kg, *n* = 12; *black column* maprotiline 10 mg/kg, *n* = 14) conditions. ^*^ *p* < 0.05, significantly differed from vehicle-injected UCMS mice. Data are expressed as mean ± SEM.

**Fig. 4:**
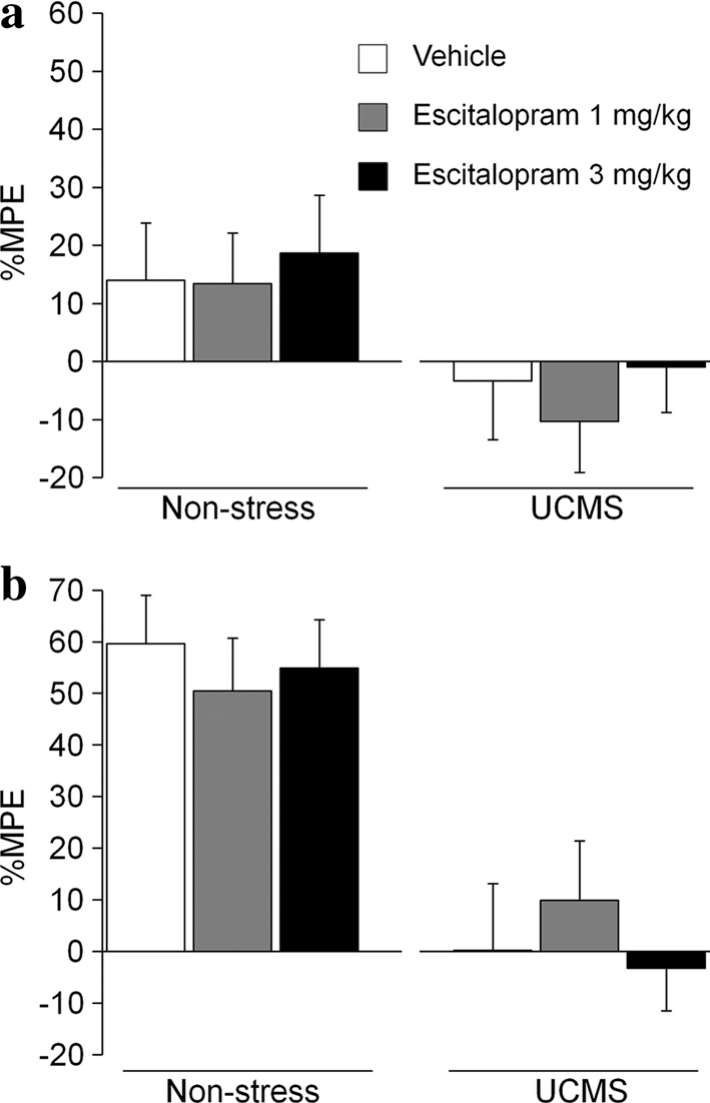
Effects of escitalopram on the antinociceptive effect of morphine under the UCMS condition. The effects of escitalopram on the thermal pain sensitivity (**a**) and the antinociceptive effect of morphine (3 mg/kg; **b**) in a hot-plate test under the UCMS (*white column* vehicle, *n* = 11; *gray column* escitalopram 1 mg/kg, *n* = 12; *black column* escitalopram 3 mg/kg, *n* = 10) and non-stress (*white column* vehicle, *n* = 12; *gray column* escitalopram 1 mg/kg, *n* = 11; *black column* escitalopram 3 mg/kg, *n* = 9) conditions. Data are expressed as mean ± SEM.

We further examined whether pretreatment with maprotiline (a noradrenalin reuptake inhibitor) or escitalopram (a serotonin reuptake inhibitor) affects the reduced antinociceptive effect of morphine (3 mg/kg) on mice that had experienced UCMS (Figs. 3, 4).

Pretreatment with maprotiline [3 and 10 mg/kg, intraperitoneal (i.p.)] itself did not exert any antinociceptive effects under either the UCMS or non-stress condition (ANOVA: UCMS, *F*_2,35_ = 0.26, *p* = 0.77; non-stress, *F*_2,37_ = 0.43, *p* = 0.65: Fig. 3a). The thermal antinociceptive effects of morphine were significantly affected by pretreatment with maprotiline under the UCMS condition (ANOVA: *F*_2,35_ = 7.20, *p* = 0.0024: Fig. 3b). The reduced thermal antinociceptive effect of 3 mg/kg morphine under the UCMS condition was significantly ameliorated by pretreatment with 10 mg/kg maprotiline (*p* < 0.05, Sidak’s multiple comparisons post hoc test). Pretreatment with maprotiline did not affect the antinociceptive effects of morphine (3 mg/kg) under the non-stress condition (ANOVA: *F*_2,37_ = 0.04, *p* = 0.96: Fig. 3b).

Pretreatment with escitalopram (1 and 3 mg/kg, i.p.) itself did not exert any antinociceptive effects under either the UCMS or non-stress condition (ANOVA: UCMS, *F*_2,30_ = 0.31, *p* = 0.74; non-stress, *F*_2,29_ = 0.08, *p* = 0.92: Fig. 4a). In contrast to the case of maprotiline, pretreatment with escitalopram did not affect the antinociceptive effects of morphine (3 mg/kg) under the UCMS and non-stress conditions (ANOVA: UCMS, *F*_2,30_ = 0.36, *p* = 0.70; non-stress, *F*_2,29_ = 0.23, *p* = 0.79: Fig. 4b).

## Discussion

The present study used mice that had experienced UCMS to demonstrate the reduced antinociceptive effects of morphine under the chronic stress condition. It has been reported that chronic stress attenuates the antinociceptive effect of morphine. Specifically, da Silva Torres et al. [4] showed that chronic restraint stress attenuated the antinociceptive effect of morphine in rats. Girardot and Holloway [5] showed that chronic cold-water swim stress reduced the analgesic effect of morphine in rats. The present study has added UCMS to the list of chronic stressors that attenuate morphine-induced analgesia.

In the present study, UCMS did not affect basal thermal pain sensitivity in a hot-plate test, although several previous studies reported a facilitative or suppressive influence of chronic stress on pain sensitivity [15–18]. Imbe et al. [16] reported that chronic restraint stress induced hyperalgesia in a tail flick test. Pinto-Ribeiro et al. [17] found reduced nociception in rats submitted to a chronic unpredictable stress paradigm using a tail-flick test. Shi et al. [18] reported increased thermal and mechanical nociceptive thresholds in rats exposed to UCMS using a hot plate test and a von Frey test, respectively, although their procedure for UCMS differed from ours. These findings suggest that the effects of chronic stress on pain sensitivity depend on the stress-inducing procedures. Further studies are required to elucidate the distinct morphological and functional changes in the central nervous system caused by each stress-induction procedure.

Unlike the case of morphine, the antinociceptive effect of tramadol was not reduced under the UCMS condition. In the context of previous reports that tramadol has inhibitory effects on noradrenaline and serotonin transporters in addition to its agonistic effect on opioid receptors [12–14], this result suggests the important role of inhibitory effects on the transporters in the antinociceptive effect of tramadol under UCMS condition. Thus, we examined the effects of pretreatment with noradrenaline and serotonin transporter inhibitors and found that pretreatment with a noradrenaline reuptake inhibitor but not a serotonin reuptake inhibitor ameliorated the reduced antinociceptive effect of morphine under the UCMS condition. Doses of serotonin transporter inhibitor (escitalopram; 1, 3 mg/kg) used in the present study are thought to be sufficient for behavioral experiments [19]. Thus, these results suggest that the reduced antinociceptive effect of morphine under the UCMS condition may be due to the down-regulation of noradrenergic transmission. In this context, Chen et al. [20] reported that chronic social defeat stress increased the expression of noradrenalin transporter mRNA and protein in the locus coeruleus (LC), which supposedly down-regulated noradrenergic transmission. Additionally, the electrophysiological study conducted by Bravo et al. [21] showed that chronic mild stress induced reduction in noradrenergic transmission in the LC.

It is thought that chronic pain itself also constitutes chronic stress. It has been reported that chronic pain induces dysfunction in the noradrenergic transmission in the LC of rats in the neuropathic pain model [22]. Combined with a series of previous findings, the present results suggest that compounds having both an agonistic effect on opioid receptors and an inhibitory effect on noradrenaline transporters, or a combination of opioids and noradrenaline reuptake inhibitors, may be effective in the treatment of patients suffering from chronic pain. In support of this notion, antidepressants with an inhibitory effect on noradrenaline transporters (e.g., serotonin–noradrenaline reuptake inhibitors (SNRIs), tricyclic antidepressants) have been reported to be more effective than selective serotonin reuptake inhibitors (SSRIs) for the treatment of chronic pain [23].

## Conclusions

The antinociceptive effects of morphine but not tramadol were reduced under the UCMS condition. Pretreatment with a noradrenaline transporter inhibitor but not a serotonin transporter inhibitor ameliorated the reduced antinociceptive effect of morphine under the UCMS condition. These results suggest that activation of the noradrenergic but not the serotonergic system may ameliorate the reduced antinociceptive effect of morphine under conditions of chronic stress.

## Methods

### Animals

Male BALB/c mice (Japan SLC, Hamamatsu, Japan) were used. The mice were maintained at a constant ambient temperature (23 ± 1°C) under a 12/12-h light/dark cycle with food and water available ad libitum. Three mice were bred in one cage (17 × 35 cm cage). All experiments were performed with the approval of the Institutional Animal Care and Use Committee at Hokkaido University.

### Drugs

Tramadol hydrochloride was gifted by Nippon Shinyaku Co., Ltd (Kyoto, Japan); morphine hydrochloride was purchased from Takeda Pharmaceutical Company, Ltd. (Osaka, Japan); maprotiline hydrochloride was purchased from Wako Pure Chemical Ind., Ltd (Osaka, Japan); and escitalopram oxalate was purchased from SIGMA Chemical Co. (St. Louis, MO).

### Unpredictable chronic mild stress (UCMS)

As shown in Table 1, mice experienced one of the stressors each day during a 5-week period (Table 1). The following stressors were applied: cage tilting (45°), damp bedding (200-ml water/cage), food deprivation, food and water deprivation, lights on overnight, small cage (change in size to 12.5 × 20 cm), cage-mate shuffle, lights off during the day, and cage exchange (changing the cage to one used by other mice).

**Table 1:** Procedures and schedule of UCMS

	Stressor	Duration (h)	Day of application
1	Cage tilting (45°)	14	1, 9, 17, 27
2	Damp bedding	24	2, 14, 21, 30, 34
3	Food deprivation	24	3, 15, 20, 28, 33
4	Food and water deprivation	14	6, 12, 24
5	Lights on overnight	24	4, 19, 25, 32
6	Small cage	24	7, 18, 29, 35
7	Cage mate shuffle	24	5, 13, 31
8	Lights off during the day	24	8, 16, 22
9	Cage exchange	24	10, 23
10	Non stress		11, 26

### Antinociceptive test

A hot-plate test was performed according a slightly modified version of the method developed by Woolfe and MacDonald [24]. A commercially available apparatus consisting of an acrylic resin cylinder (φ20 × 25 cm, diameter × height) and a thermo-controlled aluminum plate (Model MK-350HC, Muromachi Kikai Co., Tokyo, Japan) was used. Mice were placed on a 52 ± 0.5°C hot plate, and latencies to hind-paw licking or jumping were recorded with a cut-off time of 60 s. The analyses of dose-dependency were conducted by the cumulative dose–response schedule [25], in which the 3 doses of drugs are administered to the same mouse and hot-plate tests was performed 4 times (pre and after drug injections) in the present study. Morphine was repetitively administered to the mouse by i.p. injection at doses of 1, 2, and 7 mg/kg, for cumulative doses of 1, 3, and 10 mg/kg, respectively. Tramadol was repetitively administered to the mouse by i.p. injection at doses of 10, 20, and 70 mg/kg, for cumulative doses of 10, 30, and 100 mg/kg, respectively. Hot-plate tests were performed 30 min after each injection. Each injection was administered immediately after the test. In the analyses using noradrenalin or serotonin transporter inhibitors, maprotiline (3, 10 mg/kg, i.p.) or escitalopram (1, 3 mg/kg, i.p.) was administered 30 min before the injection of morphine (3.0 mg/kg). Each datum regarding latency was converted to the percent of the maximal possible effect (%MPE) according to the following formula:

$$\% {\text{MPE }} = \, ({\text{post-drug latency}} - {\text{pre-drug latency}})/({\text{cut-off time }}{-}{\text{ pre-drug latency}}) \, \times 100\%.$$

### Statistical analyses

Antinociceptive effects were statistically evaluated by ANOVA followed by the Sidak’s multiple-comparisons post hoc test using GraphPad Prism v.6.00 (GraphPad Software, San Diego, CA). *P* values <0.05 were considered to indicate statistical significance.

## Abbreviations

ANOVA: analysis of variance
i.p.: intraperitoneal
LC: locus coeruleus
MPE: maximal possible effect
UCMS: unpredictable chronic mild stress
